# A 2-year longitudinal study examining the change in psychosocial factors under the COVID-19 pandemic in Japan

**DOI:** 10.1038/s41597-024-03125-2

**Published:** 2024-05-28

**Authors:** Nagisa Sugaya, Tetsuya Yamamoto, Chigusa Uchiumi

**Affiliations:** 1 Occupational Stress and Health Management Research Group, National Institute of Occupational Safety and Health, Kawasaki, Japan; 2 Graduate School of Technology, Industrial and Social Sciences, Tokushima University, Tokushima, Japan

**Keywords:** Human behaviour, Psychology and behaviour

## Abstract

To examine changes in individuals’ psychosocial variables (e.g., psychological distress, social isolation, and alcohol use) during the prolonged COVID-19 pandemic, a two-year longitudinal survey was conducted at approximately one-year intervals between May 2020 and May 2022, after the first COVID-19-related state of emergency was announced in Japan. The online survey was conducted on May 11-12, 2020 (Phase 1), June 14–20, 2021 (Phase 2), and May 13–30, 2022 (Phase 3). The survey in Phase 1 was conducted during the first emergency declaration period, the survey in Phase 2 was conducted during the third emergency declaration period, and the survey in Phase 3 was conducted at a time when there was no state of emergency but many COVID-19 positive cases. Notably, 3,892 participants responded to all three surveys. In addition to psychosocial inventories often used worldwide, survey items included lifestyle and stress management indicators related to COVID-19 and various sociodemographic items including occupation (e.g., healthcare workers) or income, history of medical treatment for mental problems, severe physical illnesses, and COVID-19.

## Background & Summary

Coronavirus disease 2019 (COVID-19) broke out in December 2019 and spread rapidly worldwide. Although the World Health Organization (WHO) announced the end of the emergency in May 2023, as of August 2023, there were still cases of infection in many parts of the world^1^. To deter its spread, many countries have repeatedly implemented lockdowns, restricting people’s movements and temporarily closing services. However, while these lockdowns have been effective in preventing the spread of the disease, they have also caused significant economic hardships and emotional distress^2,3^.

In Japan, four states of emergency were declared between 2020 and 2021 to combat the COVID-19 outbreak. While many countries were in lockdown with penalties for violations, Japan’s COVID-19 measures were characterized by the government’s request to refrain from leaving the house, except in an emergency, temporary closure of some businesses, and no penalties for violations. As the declaration of the states of emergency in Japan was a “request” by the government, it did not prohibit people from going out or meeting with others. Despite this, even the mild lockdown^4^ in Japan affected people’s lives in various ways, including lifestyle changes due to teleworking and online classes, as well as economic hardship due to reduced income and unemployment. We have previously reported how Japanese citizens experienced severe psychological stress, depression, anxiety, loneliness, and social isolation during the state of emergency^4–8^. In addition, several previous studies have suggested that not all psychosocial variables may have changed uniformly during the pandemic. For example, no improvements were observed in severe social isolation or loneliness among the Japanese population^7^ between the two survey phases, although psychological distress improved significantly and depression decreased slightly.

Therefore, long-term studies are needed to identify the actual changes in psychosocial conditions during prolonged pandemics. To examine changes in psychosocial variables, we conducted a longitudinal survey at approximately one-year intervals during the two years after the first state of emergency for COVID-19 in individuals who experienced repeated states of emergency.

## Methods

### Participants and data collection

The online survey was conducted on May 11-12, 2020 (Phase 1), June 14–20, 2021 (Phase 2), and May 13–30, 2022 (Phase 3). Phase 1 was undertaken during the first state of emergency, while Phase 2 was carried out during the third state of emergency. The online survey in Phase 1 was conducted among residents in seven prefectures in which the state of emergency was declared relatively early (Tokyo, Kanagawa, Osaka, Saitama, Chiba, Hyogo, and Fukuoka prefectures) to accurately assess its impact. The inclusion criteria were as follows: (a) residents of the seven prefectures, and (b) aged 18 years or older. The exclusion criteria were as follows: (a) under 18 years of age, (b) high school students, and (c) living outside the seven prefectures. Phase 1 had 11,333 participants, with the number of participants from each prefecture based on the ratio of the number of people residing in each prefecture (Tokyo: n = 2783, 24.6%; Kanagawa: n = 1863, 16.4%; Osaka: n = 1794, 15.8%; Saitama: n = 1484, 13.1%; Chiba: n = 1263, 11.1%; Hyogo: n = 1119, 9.9%; Fukuoka: n = 1027, 9.1%). In Phase 2, residents living in Kanagawa, Saitama, and Chiba prefectures, where the emergency declaration did not apply, were excluded from the survey, and 4,592 residents of Tokyo, Osaka, Hyogo, and Fukuoka participated in the follow-up survey. In Phase 3, an additional follow-up survey was conducted with some participants who had participated in Phase 2 (N = 3892).

Study participants were recruited through Macromill Inc. (Tokyo, Japan), a global marketing research company. The company has more than 1.3 million registered members from all prefectures in Japan, with diverse characteristics (e.g., both genders, a wide range of age groups, and occupational statuses). The online survey system automatically eliminated duplicate responses from single respondents. A recruitment invitation was sent via e-mail to approximately 80,000 registered respondents living in the target area, and data were collected online. Upon receiving the link, participants completed an online survey voluntarily and anonymously, after providing informed consent online. Participants were given a clear explanation of the survey procedures and the release of data in a form that did not identify individuals and had the option to discontinue or terminate the survey at any time without providing a reason. Except for the default items provided by Macromill (gender, age, occupation, household income, marital status, and presence of children), the survey format did not allow participants to proceed to the next page if there were unanswered items. In addition, all participants were awarded Macromill points that they could exchange for prizes or cash.

This study was approved by the Research Ethics Committee of the Graduate School of Social and Industrial Science and Technology at Tokushima University (Approval No. 212). This study was conducted in accordance with the ethical standards of the 1964 Declaration of Helsinki and its amendments.

### Measurements

#### Sociodemographic data

Sociodemographic information, including age, gender, employment status, marital status, and household income, was collected from participants. To discuss groups assumed to be vulnerable to lockdown in previous studies in the early stages of the pandemic^9–12^, information was collected on whether the individual or family member was a healthcare worker and whether the individual was currently or had previously been treated for mental health problems, severe physical illnesses, or COVID-19.

#### Psychological distress

The Japanese version of the Kessler Psychological Distress Scale-6 (K6)^13^, a nonspecific psychological stress scale comprising six items, was used to measure psychological distress over the past 30 days. Each question was rated on a scale of 0 (never) to 4 (always), with total scores ranging from 0 to 24. The K6 is regarded as an ideal instrument for screening mental disorders in population-based health surveys because of its high accuracy and brevity^13–15^.

We adopted a threshold of five points commonly used to screen for mild-to-moderate mood/anxiety disorders^16^. Scores ranging from 5 to 12 indicated mild-to-moderate psychological distress. This is the optimal lower threshold for screening for moderate psychological distress^16^. Mild-to-moderate psychological distress is considered because of the associated risk of progression to more severe disability, as well as current distress and disability^17^. A threshold score of 13 has been traditionally used in previous studies^14,18^. A score of ≥13 was defined as serious psychological distress. Additionally, a score of ≤4 was defined as no or low psychological distress.

#### Depressive symptoms

The Japanese version of the Patient Health Questionnaire-9 (PHQ-9)^19^ was used to assess depression. Participants reported depressive symptoms for the past 4 weeks on a scale of 0 (not at all) to 3 (almost daily)^20^.

A cutoff score of 10 or higher, as recommended by previous research, indicates a high probability of major depression^19^. The PHQ-9 has been used internationally as a screening scale for depression^21^, with high reliability and validity^19^.

#### Loneliness

Loneliness was measured using the Japanese version of the UCLA Loneliness Scale version 3 (UCLA-LS3)^22^, consisting of 10 items ranging from 1 (never) to 4 (always). The total score ranged from 10 to 40, with higher scores indicating greater loneliness^23^. The UCLA-LS3 has high reliability and validity and is used internationally as a scale to measure loneliness^24–26^.

#### Social network

Social networks were assessed by using the Japanese version of the Lubben Social Network Scale (LSNS-6)^27^. The LSNS-6 is a shortened version of the Lubben Social Network Scale^28^, and it includes items on the network size of relatives and friends who provide emotional and instrumental support. The LSNS-6 consists of three items related to family networks and three items related to friendship networks. The number of people in the network was calculated on a 6-point scale (0 = none; 1 = 1 person; 2 = 2 persons; 3 = 3–4 persons; 4 = 5–8 persons; 5 = 9 or more persons) for each item^29^. Total scores ranged from 0 to 30, with higher scores indicating greater social networks and scores below 12 indicating social isolation.

#### Subjective happiness

Subjective happiness was assessed using the Japanese version of the Subjective Happiness Scale (SHS)^30^, a 4-item global subjective happiness scale. The response format is a 7-point Likert scale. One composite score is computed by averaging the responses to the four items, according to the reverse coding of the fourth item. The scores range from 1 to 7, with higher scores indicating greater well-being^31^.

#### Physical symptoms

The Japanese version of the Somatic Symptom Scale-8 (SSS-8) was used to assess the burden of physical symptoms^32^. The SSS-8 consists of eight items that assess the following physical symptoms: stomach or bowel problems; back pain; pain in the arms, legs, or joints; headache; chest pain or shortness of breath; dizziness; fatigue or low energy; and sleep disturbances. These items comprise four symptom domains: gastrointestinal, pain, cardiopulmonary, and fatigue. Participants reported the extent to which each symptom had bothered them in the past 7 days on a scale of 0 to 4 (0 = not at all; 1 = a little bit; 2 = somewhat; 3 = quite a bit; 4 = very much)^33^.

#### Alcohol use

Alcohol use was assessed using the Japanese version of the Alcohol Use Disorders Identification Test (AUDIT)^34^. As the AUDIT identifies the presence or absence of alcohol-related problems based on the past one year of alcohol use, the adoption of the AUDIT in a survey conducted every other year is optimal. The test consists of 10 items across three domains: hazardous alcohol use, dependent symptoms, and harmful alcohol use (three, three, and four items, respectively). Each item is scored on a scale of 0 to 4. The lowest AUDIT score was 0, and the highest score was 40. Higher scores indicated a higher likelihood and severity of hazardous drinking, harmful drinking, and alcohol dependence. Based on the WHO AUDIT cutoff criteria and the Japanese Ministry of Health, Labour and Welfare health guidance^35,36^, scores of 8–14 and 15 or higher were categorized into the hazardous drinking group and potential alcohol dependence group, respectively. Participants who scored 7 or less were placed in the no alcohol problem group.

#### Lifestyle, coping behavior, and stressors related to the COVID-19 pandemic

With extensive reference to the literature on the COVID-19 pandemic^9,10,12,37,38^, we developed eight lifestyle and coping behavior items, and seven stressors were assumed to be associated with the COVID-19 pandemic (refer to Yamamoto *et al*.^39^). We asked participants to rate the frequency of implementation and their experience of these items from the start of the state of emergency (Phases 1 and 2) or the last 30 days (Phase 3), to the time of the survey, on a scale of 1 (not at all) to 7 (extremely). The details of these items have been described in our previously published articles.

## Data Records

Data records are available in XLSX format from the Open Science Framework platform, together with the questionnaire description file^39^. The datasets were anonymized to remove personal information. The abbreviation guidelines for variable names are also included in the questionnaire description file.

## Technical Validation

### Verification of the suitability of the timing of the survey

We examined the suitability of the timing of the survey to investigate the impact of a prolonged pandemic considering the spread of infection and social conditions.

**Fig. 1:**
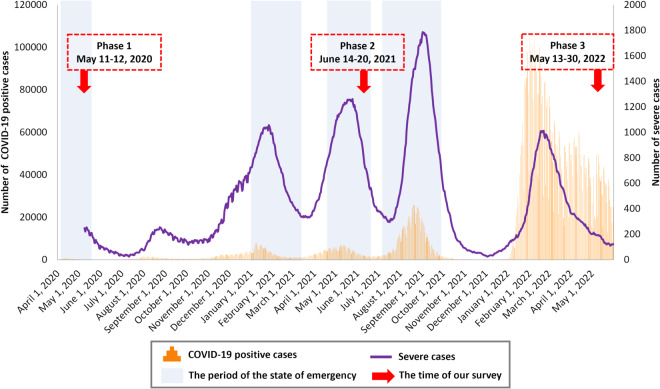
Change in the numbers of newly confirmed COVID-19-positive cases and severe cases in Japan. Newly confirmed COVID-19 positive cases: The number of newly confirmed cases is calculated by summing the number of cases published through press releases, including recurrent positive cases, by each jurisdiction. Severe cases: As a rule, severe cases are defined as those meeting one of the following conditions: (1) connected to a mechanical ventilator, (2) on ECMO, or (3) treated in the ICU (or a similar facility). However, certain jurisdictions may use other definitions.

The first survey (Phase 1, May 11-12, 2020) was conducted during the first emergency declaration period, the second survey (Phase 2, June 14-20, 2021) was conducted during the third emergency declaration period, and the third survey (Phase 3, May 13–30, 2022) was conducted during a period of no emergency and relatively few severe cases, but with the presence of many COVID-19-positive cases (Fig. 1)^40^. The emergency declaration in Japan was the government’s request to refrain from leaving the house except in an emergency and temporary closure of some businesses (e.g., restrictions on the use of facilities that attract large numbers of people), and there were no penalties for violations. The investigation dates of Phases 1 and 2 were toward the ending of the states of emergency, when the effects of lifestyle changes might have been amplified. This study will enable us to observe changes in psychosocial variables across long-term periods, as well as changes in these variables due to changes in social conditions, such as the declaration of a state of emergency. As such, it will provide useful information in considering when and which factors to intervene therewith during a prolonged pandemic.

### Verification of the suitability of exposure factors

We verified the suitability of the assumption that the prolonged COVID-19 pandemic was an exposure factor that placed a long-term burden on mental health compared to studies conducted before the pandemic.

In our dataset (N = 3892), the annual household incomes of 813 participants in Phase 1, 742 participants in Phase 2, and 686 participants in Phase 3, as well as the AUDIT scores of 4 participants (all under 20 years of age) in Phase 2 were not provided; there were no missing data for other variables. Regarding the AUDIT, the survey company’s rules prevent participants under 20 years of age from responding to the AUDIT items, given that drinking alcohol under the age of 20 is legally prohibited in Japan. Table 1 shows the survey participants’ sociodemographic characteristics.

**Table 1:** Sociodemographic characteristics of the surveys.

Sociodemographic indexes	Phase 1		Phase 2		Phase 3	
(Total: N = 3892)	N (%)		N (%)		N (%)	
Sex (Female)	1813	(46.6%)	—		—	
Age						
*18–29*	273	(7.0%)	224	(5.8%)	186	(4.8%)
*30–49*	1575	(40.5%)	1514	(38.9%)	1448	(37.2%)
*50–64*	1395	(35.8%)	1454	(37.4%)	1510	(38.8%)
*≥65*	649	(16.7%)	700	(18.0%)	748	(19.2%)
Occupation						
*Employed*	2693	(69.2%)	2677	(68.8%)	2693	(69.2%)
*Homemaker*	585	(15.0%)	594	(15.3%)	566	(14.5%)
*Student*	46	(1.2%)	27	(0.7%)	19	(0.5%)
*Unemployed*	443	(11.4%)	480	(12.3%)	564	(14.5%)
*Other*	125	(3.2%)	114	(2.9%)	50	(1.3%)
Marital status (Married)	2489	(64.0%)	2500	(64.2%)	2490	(64.0%)
Presence of child (Yes)	2175	(55.9%)	2226	(57.2%)	2224	(57.1%)
Annual household income (JPY)						
*<2.0 million*	223	(5.7%)	247	(6.3%)	333	(8.6%)
*2.0–3.9 million*	737	(18.9%)	765	(19.7%)	756	(19.4%)
*4.0–5.9 million*	775	(19.9%)	802	(20.6%)	754	(19.4%)
*6.0–7.9 million*	525	(13.5%)	515	(13.2%)	517	(13.3%)
*> = 8.0 million*	819	(21.0%)	821	(21.1%)	846	(21.7%)
*Unknown*	813	(20.9%)	742	(19.1%)	686	(17.6%)
Healthcare worker (Yes)						
*Self*	214	(5.5%)	236	(6.1%)	215	(5.5%)
*Family*	307	(7.9%)	268	(6.9%)	247	(6.3%)
Treatment of severe physical diseases (Yes)						
*Current*	191	(4.9%)	187	(4.8%)	208	(5.3%)
*Past*	321	(8.2%)	256	(6.6%)	330	(8.5%)
Treatment of mental problems (Yes)						
*Current*	208	(5.3%)	198	(5.1%)	209	(5.4%)
*Past*	428	(11.0%)	315	(8.1%)	318	(8.2%)
Treatment of COVID-19 (Yes)						
*Current*	9	(0.2%)	3	(0.1%)	12	(0.3%)
*Past*	11	(0.3%)	13	(0.3%)	115	(3.0%)

In the comparisons of psychosocial and physical variables between phases by the repeated measures analysis of variance and paired-samples *t*-test (only the AUDIT), there were significant differences between phases in all variables except “Healthy sleep habits” and the AUDIT score (Table 2). Regarding the UCLA-LS3, the SHS, “Healthy eating habits,” “Altruistic preventive behavior,” “Deterioration of household economy,” “Frustration,” and “COVID-19-related sleeplessness,” results did not exceed the lower limit of “small effect size” (*η*^2^ ≥ 0.010), although there were statistically significant effects of phase in these variables. While many indicators showed improvement from Phase 1 to Phase 3, there were increases in the UCLA-LS3 scores and “Deterioration of relationship with familiar people,” as well as decreases in the LSNS-6 scores, “Exercise,” “Favorite activity,” and “Online interaction with familiar people.”

**Table 2:** Change of each variable during three phases.

	Phase 1		Phase 2		Phase 3		*F*	*p*	*η*^2^
	Mean (SD)		Mean (SD)		Mean (SD)				
K6	5.1	(5.3)	4.0	(5.2)	3.9	(5.4)	167.66	<0.001	0.041
PHQ-9	4.4	(5.4)	3.9	(5.4)	3.8	(5.4)	44.05	<0.001	0.011
UCLA-LS3	23.6	(5.8)	24.0	(5.9)	23.9	(6.0)	21.48	<0.001	0.005
LSNS-6	10.0	(6.1)	9.4	(6.0)	9.3	(6.0)	42.94	<0.001	0.011
SHS	4.4	(1.0)	4.3	(1.0)	4.4	(1.1)	29.81	<0.001	0.008
SSS-8	6.3	(5.5)	5.1	(5.5)	5.1	(5.5)	187.41	<0.001	0.046
AUDIT *		—	4.4	(6.1)	4.4	(5.8)	0.67	0.501	0.011
Exercise	4.1	(1.8)	3.8	(1.9)	3.8	(1.9)	95.75	<0.001	0.024
Healthy eating habits	4.3	(1.5)	4.2	(1.6)	4.2	(1.6)	19.89	<0.001	0.005
Healthy sleep habits	4.7	(1.7)	4.7	(1.7)	4.8	(1.7)	2.21	0.111	0.001
Favorite activity	4.0	(1.6)	3.7	(1.7)	3.8	(1.7)	53.23	<0.001	0.013
Offline interaction with familiar people	3.5	(1.8)	3.3	(1.8)	3.9	(1.8)	177.67	<0.001	0.044
Online interaction with familiar people	3.1	(1.9)	2.7	(1.7)	2.8	(1.8)	90.16	<0.001	0.023
Altruistic preventive behavior	5.4	(1.7)	5.4	(1.7)	5.4	(1.7)	3.89	0.021	0.001
Optimism	4.0	(1.5)	4.2	(1.5)	4.3	(1.5)	69.17	<0.001	0.017
Deterioration of household economy	3.7	(1.8)	3.5	(1.7)	3.5	(1.7)	35.68	<0.001	0.009
Deterioration of relationship with familiar people	2.4	(1.5)	2.6	(1.6)	2.6	(1.5)	61.53	<0.001	0.016
Frustration	3.2	(1.7)	3.2	(1.7)	3.1	(1.7)	4.43	0.035	0.001
COVID-19-related anxiety	3.9	(1.7)	3.4	(1.7)	3.2	(1.6)	324.70	<0.001	0.077
COVID-19-related sleeplessness	2.5	(1.5)	2.5	(1.5)	2.4	(1.5)	5.58	0.004	0.001
Difficulties owing to the lack of daily necessities	3.5	(1.8)	2.5	(1.5)	2.5	(1.5)	684.09	<0.001	0.150
Difficulties in work or schoolwork	3.6	(2.0)	2.8	(1.7)	2.7	(1.7)	465.29	<0.001	0.107

* Because the paired-samples *t* test was applied for the AUDIT, *t* value and Cohen’s *d* were provided as the effect size.

**Fig. 2:**
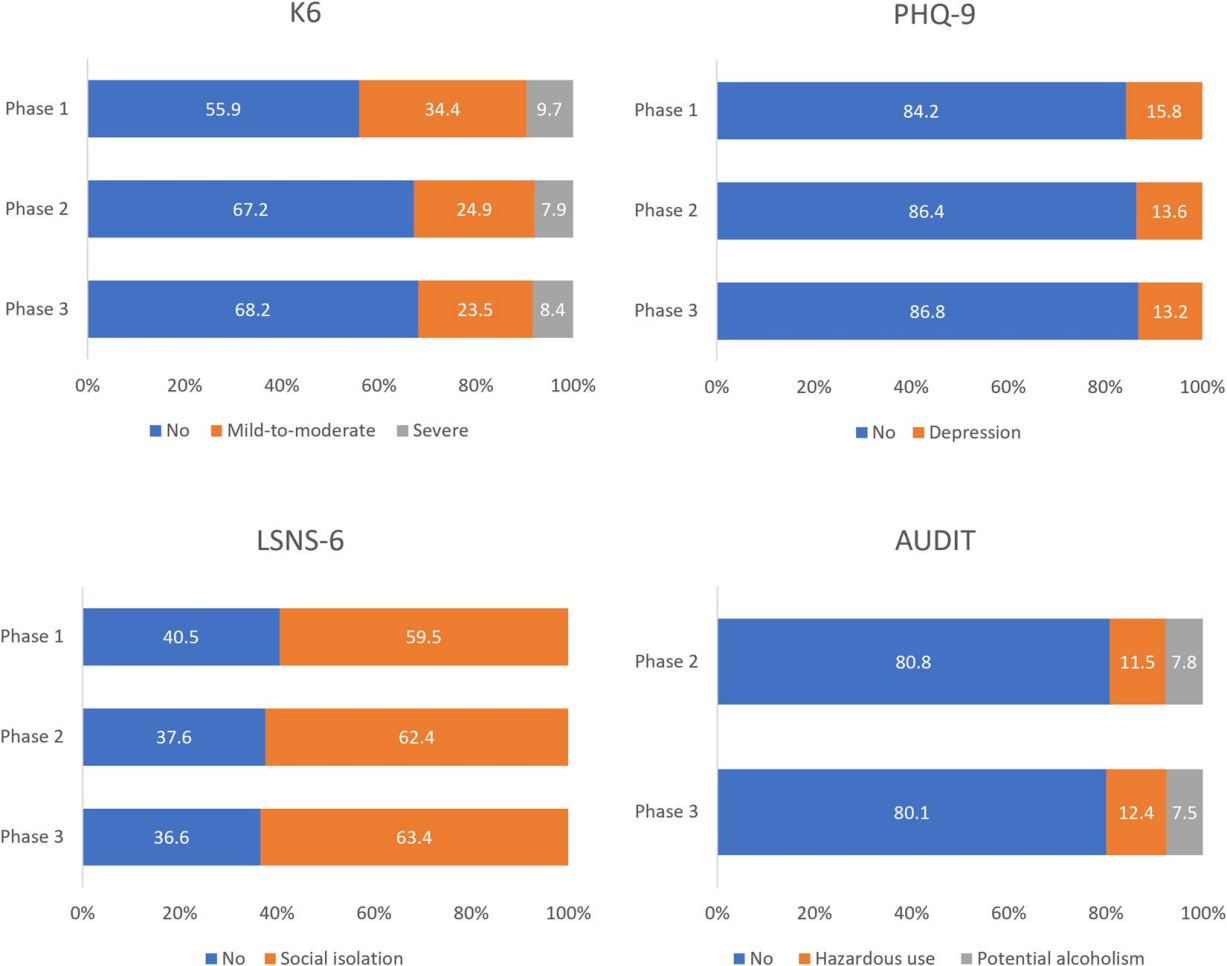
Percentage of psychosocial problems based on cutoffs for each measure.

Figure 2 shows the percentage of psychosocial problems based on the cutoff values for the K6, PHQ-9, LSNS-6, and AUDIT. For the K6, the prevalence of mild-to-moderate psychological distress (K6 score = 5–12) decreased significantly from Phase 1 to Phase 2 (Phase 1: 34.4%, Phase 2: 24.9%), whereas the prevalence of severe psychological distress (K6 score ≥ 13) changed only slightly over the 2-year period (Phase 1: 9.7%; Phase 2: 7.9%). According to data published by the Ministry of Health, Labour and Welfare in 2019 concerning the K6 in the Japanese population, 26.9% of participants had mild-to-moderate or severe psychological stress (i.e., K6 score ≥ 5)^41^ while our data showed that 44.1% and 32.8% of participants had it in Phases 1 and 2, respectively. For the PHQ-9, the prevalence of depression (PHQ-9 score ≥ 10) decreased slightly from Phase 1 to Phase 2 (Phase 1: 15.8%, Phase 2: 13.6%). In a previous survey of the general Japanese population conducted in 2013, 7.9% of participants reported a PHQ-9 score of ≥10^42^. Thus, we could conclude that psychological distress and depression did not improve sufficiently in our survey, even in Phase 3, compared with before the pandemic. For the LSNS-6, the prevalence of social isolation increased slightly from Phase 1 to Phase 2. In a previous study conducted before the pandemic^27^, the prevalence of social isolation (less than 12 points) in Japan was 19.4%, and our survey showed a much higher prevalence. For the AUDIT, there was a slight increase in hazardous users, but little change in the prevalence of potential alcoholism. Based on gender, the prevalence of potential alcoholism in men was 10.4% in Phase 2 and 10.1% in Phase 3, whereas that of hazardous alcohol use was 16.0% in Phase 2 and 17.5% in Phase 3. The prevalence of potential alcoholism in women was 4.7% in Phase 2 and 4.6% in Phase 3, and that of hazardous alcohol use was 6.2% in Phase 2 and 6.6% in Phase 3. Prior research before the pandemic showed that the prevalence of potential alcoholism in Japan was 5.2% for men and 0.7% for women, and that of hazardous alcohol use was 16.2% for men and 3.8% for women^43^. In men, the prevalence of potential alcoholism increased compared to a previous study^43^, and in women, both the prevalence of hazardous use and potential alcoholism increased, with the latter being particularly prominent.

## Usage Notes

This dataset was obtained through a large-scale survey conducted annually over a two-year period, allowing for time series analysis stratified by demographic characteristics and other factors. Two of the three time points surveyed were at the final stages of the state of emergency, a period of particular stress, and thus may be useful for detecting the effects of the emergency. In addition, several globally used measures were employed in the dataset, which facilitated comparisons with other studies.

However, this study has several limitations. First, as the data were collected through an online survey, random sampling was not conducted. Therefore, we cannot guarantee the representativeness of the sample, as it cannot be matched to the percentages of each age group or gender in each region. Compared with the actual age ratio, the data are more skewed toward the middle-aged and less toward the elderly^44^, and accordingly, the household income is higher^45^, and a higher percentage of participants were workers in terms of their employment status^46^. Therefore, it may be desirable to consider age when analyzing the data, if necessary. However, when people are encouraged to avoid unnecessary outings, online surveys can be a valuable way to assess people’s health status. Second, although it is natural that the number of past treatments for mental problems and severe physical illnesses should increase over time, it decreased in Phases 2 and 3. This may indicate that the definitions of mental problems and severe physical illnesses differed for participants in each phase, and the data on these items should be treated with caution. Third, the dropout rate of participants in this study was high; 2,831 (42.1%) of the 6,723 individuals who participated in Phase 1 did not respond in Phase 2 or 3. In addition, there were significantly more females than males among individuals who did not participate in Phase 2 or 3 (*p* < 0.001). Individuals who did not participate in Phase 2 or 3 were younger and had lower UCLA-LS3 scores and higher LSNS-6, K6, PHQ-9, and SSS-8 scores than those who participated in the three phases (*p* < 0.001). Thus, many participants with mental and physical problems may have been excluded from the dataset.

## Data Availability

No custom codes were used in this study. Microsoft Excel was used to tabulate and distribute the data.
